# Associations of sleep disorders with serum neurofilament light chain levels in Parkinson’s disease

**DOI:** 10.1186/s12883-024-03642-y

**Published:** 2024-05-01

**Authors:** Wan-Yi Qi, Yan Sun, Yun Guo, Lan Tan

**Affiliations:** 1 Department of Neurology, Qingdao Municipal Hospital, Dalian Medical University, No.5 Donghai Middle Road, Qingdao, China; 2 Department of Neurology, Qingdao Municipal Hospital, Qingdao University, Qingdao, China; 3 School of Clinical Medicine, Weifang Medical University, Weifang, 261053 China

**Keywords:** Parkinson’s disease, REM sleep behavior disorder, Excessive daytime sleepiness, Sleep, Neurofilament protein L

## Abstract

**Background:**

Sleep disorders are a prevalent non-motor symptom of Parkinson’s disease (PD), although reliable biological markers are presently lacking.

**Objectives:**

To explore the associations between sleep disorders and serum neurofilament light chain (NfL) levels in individuals with prodromal and early PD.

**Methods:**

The study contained 1113 participants, including 585 early PD individuals, 353 prodromal PD individuals, and 175 healthy controls (HCs). The correlations between sleep disorders (including rapid eye movement sleep behavior disorder (RBD) and excessive daytime sleepiness (EDS)) and serum NfL levels were researched using multiple linear regression models and linear mixed-effects models. We further investigated the correlations between the rates of changes in daytime sleepiness and serum NfL levels using multiple linear regression models.

**Results:**

In baseline analysis, early and prodromal PD individuals who manifested specific behaviors of RBD showed significantly higher levels of serum NfL. Specifically, early PD individuals who experienced nocturnal dream behaviors (*β* = 0.033; *P* = 0.042) and movements of arms or legs during sleep (*β* = 0.027; *P* = 0.049) showed significantly higher serum NfL levels. For prodromal PD individuals, serum NfL levels were significantly higher in individuals suffering from disturbed sleep (*β* = 0.038; *P* = 0.026). Our longitudinal findings support these baseline associations. Serum NfL levels showed an upward trend in early PD individuals who had a higher total RBDSQ score (*β* = 0.002; *P* = 0.011) or who were considered as probable RBD (*β* = 0.012; *P* = 0.009) or who exhibited behaviors on several sub-items of the RBDSQ. In addition, early PD individuals who had a high total ESS score (*β* = 0.001; *P* = 0.012) or who were regarded to have EDS (*β* = 0.013; *P* = 0.007) or who exhibited daytime sleepiness in several conditions had a trend toward higher serum NfL levels.

**Conclusion:**

Sleep disorders correlate with higher serum NfL, suggesting a link to PD neuronal damage. Early identification of sleep disorders and NfL monitoring are pivotal in detecting at-risk PD patients promptly, allowing for timely intervention. Regular monitoring of NfL levels holds promise for tracking both sleep disorders and disease progression, potentially emerging as a biomarker for evaluating treatment outcomes.

**Supplementary Information:**

The online version contains supplementary material available at 10.1186/s12883-024-03642-y.

## Background

Currently, early and accurate diagnosis of PD remains a challenge. Recent studies have increasingly focused on the non-motor symptoms (NMS) of Parkinson’s disease (PD) [1]. Sleep disorders are considered to be one of the most prevalent NMS of PD and cover a wide range of conditions, such as rapid eye movement (REM) sleep behavior disorder (RBD), insomnia, restless legs syndrome (RLS), obstructive sleep apnea (OSA), and excessive daytime sleepiness (EDS) [2]. RBD is a reliable clinical predictor of neurodegenerative disease. Previous studies have shown that RBD is one of the earliest sign of alpha-synucleinopathies, characterized by an intermittent loss of normal muscle tone that happens mainly in the REM sleep phase [3]. In addition, EDS is considered to be one of the earliest NMS of PD and a significant cause of disability [4, 5]. The main characteristic of EDS is the incapacity to remain awake and attentive throughout the waking hours of the day, which causes unintentional lapses into sleepiness or sleep and adversely affects safety and quality of life [6]. Sleep disorders have become a focal point of research in the field of neurodegenerative diseases. However, the precise mechanisms by which they reflect or influence the neurodegenerative process in PD remain incompletely understood. Although sleep disorders are not exclusive to PD and lack specificity across neurodegenerative conditions, they represent one of the most common NMS in PD. They sometimes precede motor symptoms in the early stages of PD and, as the disease progresses, can have a profoundly negative impact on patients’ physical function, quality of life, and overall health [7], potentially exceeding the disabling capacity of motor symptoms. Furthermore, it is important to note that sleep disorders not only serve as an early symptom of PD but also significantly contribute to the disease’s progression. By assessing patients’ risk before the typical motor symptoms of PD manifest, we can facilitate early intervention, thereby opening up new avenues for enhancing the overall health and quality of life of PD patients. Currently, the potential of sleep disorders as predictors of neuronal damage remains largely unexplored. Establishing this connection provides a novel perspective on potential alterations within the nervous system associated with early NMS.

As one of the most promising biomarkers of axonal injury, elevated blood or cerebrospinal fluid (CSF) concentrations of NfL in PD individuals were found to be associated with greater PD severity, reduced life expectancy, and an increased susceptibility to motor and cognitive impairment [8, 9]. In recent years, an increasing body of research has shown a robust correlation between serum NfL and CSF NfL and that the diagnostic accuracy of serum NfL is the same as that of CSF NfL in the discrimination between PD and atypical parkinsonian disorders (APD) [10]. Whether sleep disorders could increase the level of serum NfL has not been fully investigated.

Our research aims to determine whether sleep disorders can predict elevated serum NfL levels, aiming to establish a correlation between PD clinical symptoms and the pathological alterations stemming from neuronal damage. Early detection and diagnosis of PD are critical for effective disease management. By identifying sleep disorders early and monitoring NfL levels, we can evaluate patients’ susceptibility before noticeable PD symptoms become prominent, enabling early intervention. In addition, the use of NfL as a biomarker for monitoring sleep disorders, tracking disease progression and evaluating treatment efficacy can help to make timely adjustments to treatment regimens in order to slow down disease progression. This approach not only optimizes the treatment and management of PD patients but also provide new therapeutic targets for specific sleep disorders, opening up new possibilities for improving the overall health and quality of life of individuals with PD. We have conducted baseline and longitudinal analyses of individuals belonging to three distinct categorical groups (early PD, prodromal PD and HCs) to examine the baseline and longitudinal connections between sleep disorders and serum NfL levels. Given the gender differences in sleep disorders, we further conducted a subgroup analysis stratified by gender.

## Materials and methods

### Participants from the PPMI cohort

Data from the Parkinson’s Progression Markers Initiative (PPMI) database was utilized in this study. The management of the database was administered by the PPMI Bioinformatics Core, which belongs with the Laboratory of NeuroImaging (LONI) at the University of Southern California. Standardized protocols and data were available for download on the website (http://www.ppmi-info.org). The purpose of this ongoing longitudinal, multicentered cohort study is to find biomarkers for PD progression in order to enhance knowledge of the disease’s underlying causes and to develop essential tools that may increase the chances of success in therapy trials for PD. The PPMI received approval from the institutional review boards of all participating sites, and each participant gave written, informed permission [11]. According to the inclusion criteria of the PPMI, our cohort consisted of individuals aged 30 years or older, including early PD (participants who were diagnosed with PD during a span of two years but did not receive or were not required to undergo drug treatment for PD), prodromal PD (participants who have not yet advanced to the point of being clinically diagnosed with PD, but other NMS or indications of neurodegeneration might be seen) and healthy controls (HCs). Each participant in the cross-sectional analysis completed the main sleep-related assessments (Rapid Eye Movement Sleep Behavioural Disorder Screening Questionnaire [RBDSQ] and Epworth Sleepiness Scale [ESS]), baseline serum NfL measurement, and the Montreal Cognitive Assessment (MoCA). After excluding three individuals with serious neurological conditions that could potentially impact sleep-related assessments (one with severe craniocerebral trauma and two with tumors), our study ultimately included 1113 individuals, comprising 585 PD patients, 353 prodromal PD patients, and 175 HCs. In addition, PPMI has established prodromal cohorts based on olfaction, RBD, or genetic mutation (LRRK2, GBA, SNCA, or rare genetic mutations such as Parkin or Pink1). The inclusion and exclusion criteria for these subjects can be downloaded from the website which is available online (available at http://www.ppmi-info.org) [12].

### Sleep characteristics in the PPMI cohort

Sleep characteristics in the PPMI database were assessed by the RBDSQ and ESS questionnaires, both of which are self-rating questionnaires. The RBDSQ comprises ten items to evaluate the subject’s sleeping habits. The score is calculated by adding the weighted dichotomic responses to Items 1 to 10 (YES = 1, NO = 0) [13]. An RBDSQ score greater than or equal to 6 is used as a screening criterion for probable RBD (pRBD) since a score of 6 is thought to be the ideal threshold [14]. Items 1 to 4 asked about the frequency and complexity of dreams and their associations with nocturnal motor behaviors. Item 5 asked about harming oneself or one’s bed partner. Item 6 which had four sub-items assessed nocturnal motor behavior more precisely. Items 7–9 addressed sleep disorders. Item 10 discusses neurological disorders that might interfere with sleep but were not included in our analysis. The ESS questionnaire was utilized to simulate 8 scenarios of daytime sleepiness (never = 0, modest = 1, moderate = 2, and excessive = 3), with the total score ranging from 0 to 24. And EDS was diagnosed in those patients with an ESS score of ≥ 10 [15, 16].

### Serum NfL in the PPMI cohort

Serum NfL levels of the PPMI cohort were assessed using the Simoa Human NF-light Advantage kit using Single Molecule Array technology in a completely automated SIMOA® HD-1 analyzer (Quanterix, Lexington, MA, USA) [17]. The Simoa technology provides a reliable quantitative assay of blood NfL levels [18, 19]. Detailed information on the handling of the samples may be accessed in the manual of the PPMI (http://www.ppmi-info.org).

### Statistical analysis

**Fig. 1:**
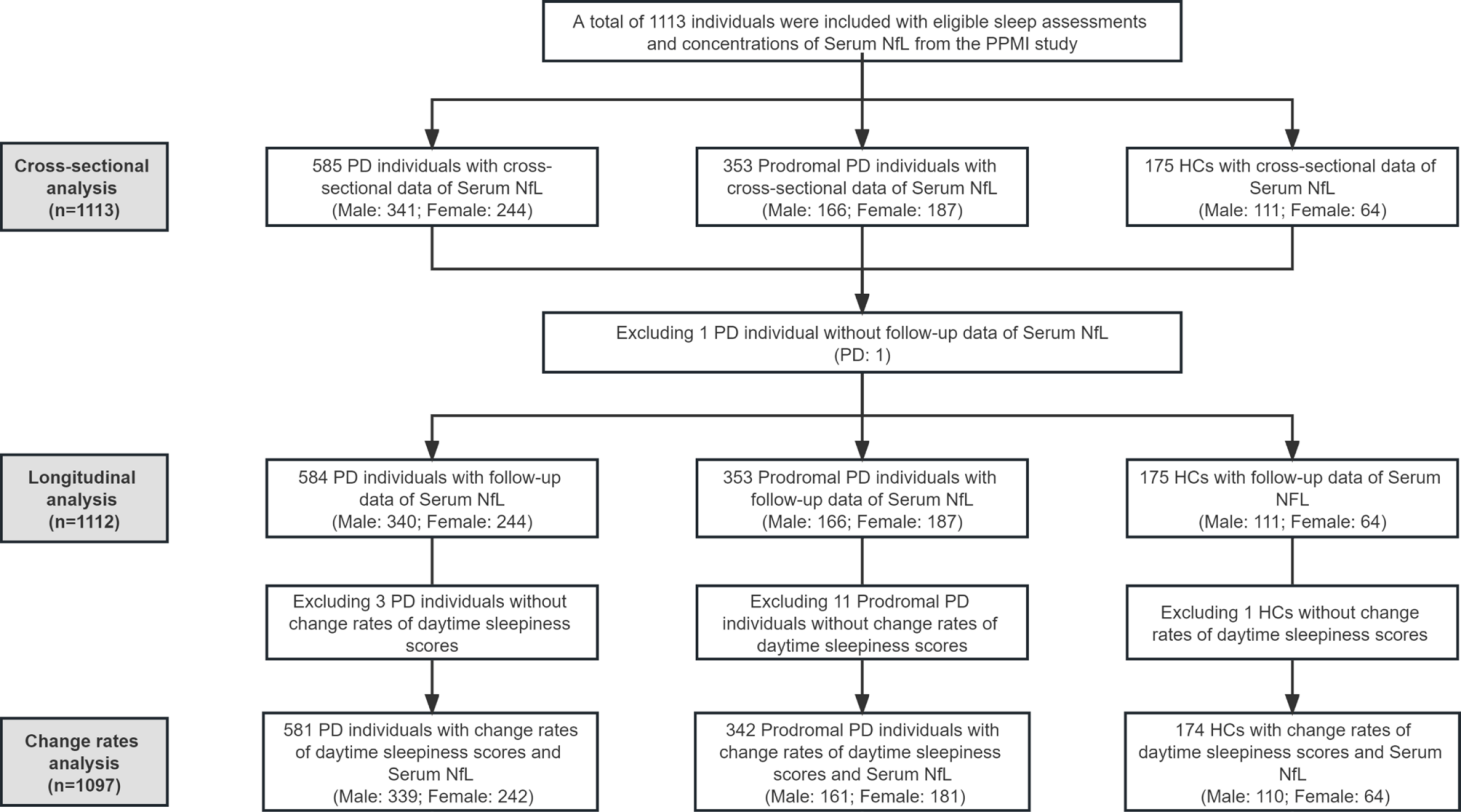
Flowchart of data analysis PPMI Parkinson’s Progression Markers Initiative, NfL Neurofilament light chain, PD Parkinson’s disease, HC healthy control

Descriptive statistical methods were used to provide a summary of the baseline characteristics of the participants. The Kruskal-Wallis test was utilized to assess the three group’s differences. Serum NfL levels were log-transformed by the “car” package in R to achieve a normal distribution (Shapiro-Wilk test > 0.05). To reduce the influence of possible extreme outliers, we defined outliers as 3 SD below or above the average. Moreover, in the PD group, disease duration was further adjusted, and in the prodromal PD group, adjustments were made for diagnoses of hyposmia, RBD, and genetic mutations. In cross-sectional analyses, after controlling for possible confounders (e.g., age, gender, and years of education), separating models of multiple linear regression were conducted for various combinations of serum NfL levels and individual sleep characteristics, using serum NfL levels as the dependent variable and the subitems of the sleep questionnaire as independent variables. Using the fitted linear mixed-effects model, the longitudinal analysis investigated the effect of baseline sleep behaviors on longitudinal serum NfL levels. The statistical model used random intercepts, time-dependent slopes, and an unstructured covariance matrix for the random effects. Additionally, the model included the interaction between time and baseline sleep disorders as predictors. In addition, after excluding individuals who did not provide the change rates of ESS scores (the sub-items of the RBDSQ questionnaire cannot be used to calculate the rate of change because they are dichotomous variables), we also calculated the average change in rates of levels of serum NfL and ESS item scores (the sim function in the “arm” package with 10,000 replicates was used for estimation) in the entire sample by linear mixed-effects models. Multiple linear regression models were used to investigate the correlations between the longitudinal changes in ESS scores and the longitudinal rates of change in serum NfL levels. All analyses were performed separately among the PD group, the prodromal PD group, and HCs. We further performed subgroup analyses by gender in these three groups. The flow chart of the main analyses is shown in Fig. 1.

## Results

### Participant characteristics

Our cross-sectional analysis analyzed the 1113 participants included at baseline, consisting of 585 PD patients, 353 prodromal PD patients, and 175 HCs. The participants’ demographic characteristics were provided in Table 1. There were no significant statistical variations noted in age between the three groups. As for the gender distribution, the female proportions were 41.71%, 52.97%, and 36.57% for PD group, prodromal PD group and HCs, respectively. In addition, all three groups of participants had an average of more than 15 years of education. Although the mean MoCA scores in the three groups were all above 25, there were statistical differences among them (*P* < 0.001). The prodromal PD group had higher total RBDSQ scores (3.39 ± 2.87) compared to both the PD group (3.27 ± 2.74) and the HC group (2.58 ± 2.14) (*P* = 0.024). The total ESS scores of PD group (6.17 ± 4.03) were higher than those of prodromal PD group (5.65 ± 3.46) and HCs (5.70 ± 3.46) (*P* = 0.294). In addition, the PD group had higher baseline levels of serum NfL (median concentration 11.9 pg/ml; range 2.76–46.6 pg/ml) compared to both the prodromal PD and HC groups (*P* = 0.005).

**Table 1:** Baseline characteristics of included participants

Variables	PD (*n* = 585)	Prodromal PD (*n* = 353)	HC (*n* = 175)	*P* values
**Age**				*P* = 0.459
Mean ± SD	61.94 ± 9.93	63.07 ± 7.52	61.11 ± 10.93	
(Minimum, maximum)	(32.20, 85.20)	(42.10, 84.30)	(31.00, 83.70)	
**Sex (n, %)**				*P* < 0.001
Male	341 (58.29%)	166 (47.03%)	111 (63.43%)	
Female	244 (41.71%)	187 (52.97%)	64 (36.57%)	
**Educate years**				*P* < 0.001
Mean ± SD	15.38 ± 3.57	16.61 ± 4.04	16.06 ± 2.84	
(Minimum, maximum)	(0.00, 26.00)	(0.00, 30.00)	(8.00, 23.00)	
**Montreal cognitive assessment**				*P* < 0.001
Mean ± SD	26.70 ± 2.84	26.70 ± 2.58	28.19 ± 1.09	
(Minimum, maximum)	(11.00, 30.00)	(11.00, 30.00)	(27.00, 30.00)	
**RBDSQ total scores**				*P* = 0.024
Mean ± SD	3.27 ± 2.74	3.39 ± 2.87	2.58 ± 2.14	
(Minimum, maximum)	(0.00, 12.00)	(0.00, 12.00)	(0.00, 11.00)	
**ESS total scores**				*P* = 0.294
Mean ± SD	6.17 ± 4.03	5.65 ± 3.46	5.70 ± 3.46	
(Minimum, maximum)	(0.00, 24.00)	(0.00, 20.00)	(0.00, 19.00)	
**Serum total NfL (pg/ml)**				*P* = 0.005
Median	11.90	11.80	10.60	
(Minimum, maximum)	(2.76, 46.60)	(3.34, 47.10)	(2.75, 51.00)	

Numbers and percentages are used to express categorical variables. Means ± SDs is used to express continuous variables

*Abbreviations* n number; SD Standard deviations; RBDSQ Rapid Eye Movement Sleep Behavioural Disorder Screening Questionnaire; ESS Epworth Sleepiness Scale

### Cross-sectional associations of sleep disorders with serum NfL levels

**Fig. 2:**
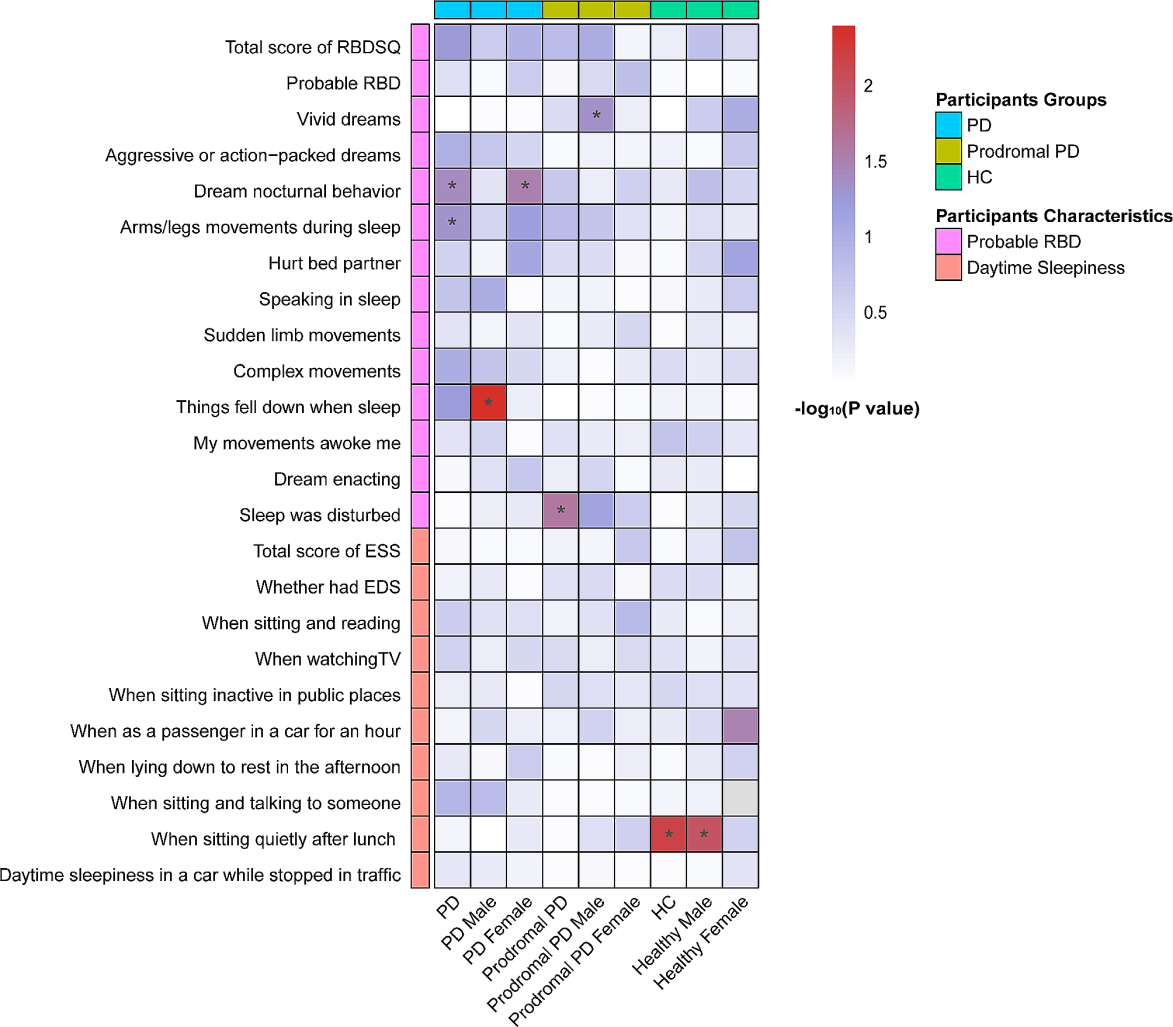
Associations of sleep disorders with serum NfL levels in cross-sectional analyses. *P < 0.05

**Fig. 3:**
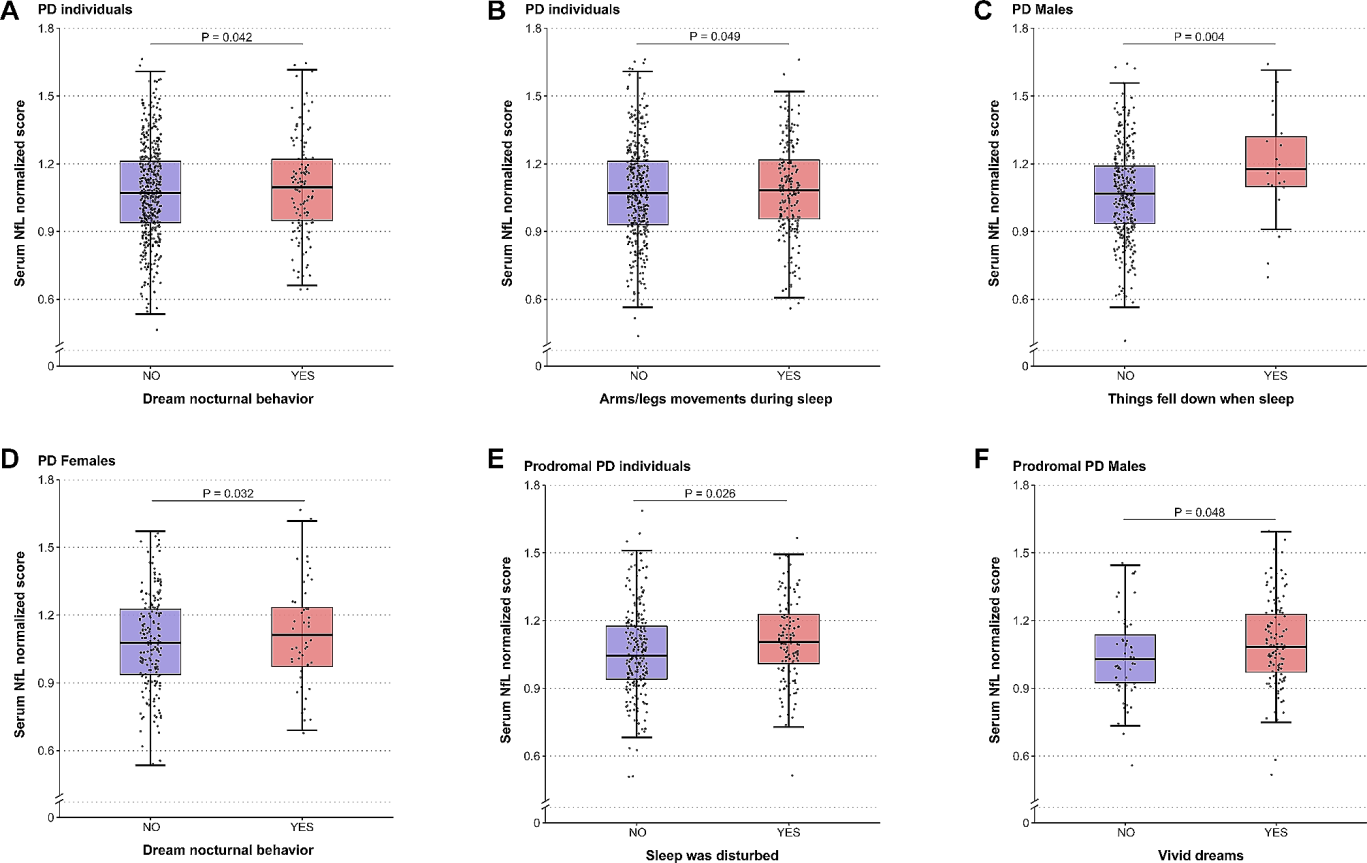
Cross-sectional associations of sleep disorders with serum NfL levels. PD individuals with dream nocturnal behavior (**A**) and arms/legs movements during sleep (**B**), PD males with things falling down when sleep (**C**), PD females with dream nocturnal behavior (**D**), prodromal PD individuals with disturbance of sleep (**E**), and prodromal PD males with vivid dreams (**F**) contribute to a higher levels of Serum NfL

The baseline associations between sleep disorders and serum NfL levels were illustrated in Fig. 2. Individuals of PD with specified RBD behaviors, including dream nocturnal behavior (*β* = 0.033; *P* = 0.042) (Fig. 3A), moving arms or legs during sleep (*β* = 0.027; *P* = 0.049) (Fig. 3B), showed significantly higher levels of serum NfL. In the subgroup analysis by gender, feeling things falling when asleep (*β* = 0.100; *P* = 0.004) showed a significant association with higher serum NfL levels in PD males (Fig. 3C); and the association for dream nocturnal behavior (*β* = 0.054; *P* = 0.032) was still significant in PD females but turned non-significant in PD males (Fig. 3D). As for EDS, in total PD participants, there was no correlation seen between daytime sleepiness and serum NfL levels (Supplementary Table S2). The gender subgroup analysis showed no significant association of the ESS scores (the total score and scores of the subitems) with serum NFL levels in two gender subgroups (Supplementary Table S2).

In prodromal PD participants, among all the RBD and ESS items, only disturbed sleep (*β* = 0.038; *P* = 0.026) (Fig. 3E), was obviously correlated with higher serum NfL levels. When stratified by gender, only the RBD item of vivid dreams (*β* = 0.052; *P* = 0.048) (Fig. 3F) was significantly correlated with higher levels of serum NfL in the male subgroup, whereas no significant correlation was found for any RBD or ESS item in the female subgroup (Supplementary Table S3 and S4).

In HCs, we found no specific RBD behaviors had significant associations with serum NfL levels (Supplementary Table S5). In terms of the ESS, an increased possibilities of daytime sleepiness when sitting quietly after lunch was significantly correlated higher levels of serum NfL, but only in the entire cohort of HCs and the male subgroup thereof (Supplementary Table S6).

### Associations of baseline sleep disorders with longitudinal serum NfL levels

**Fig. 4:**
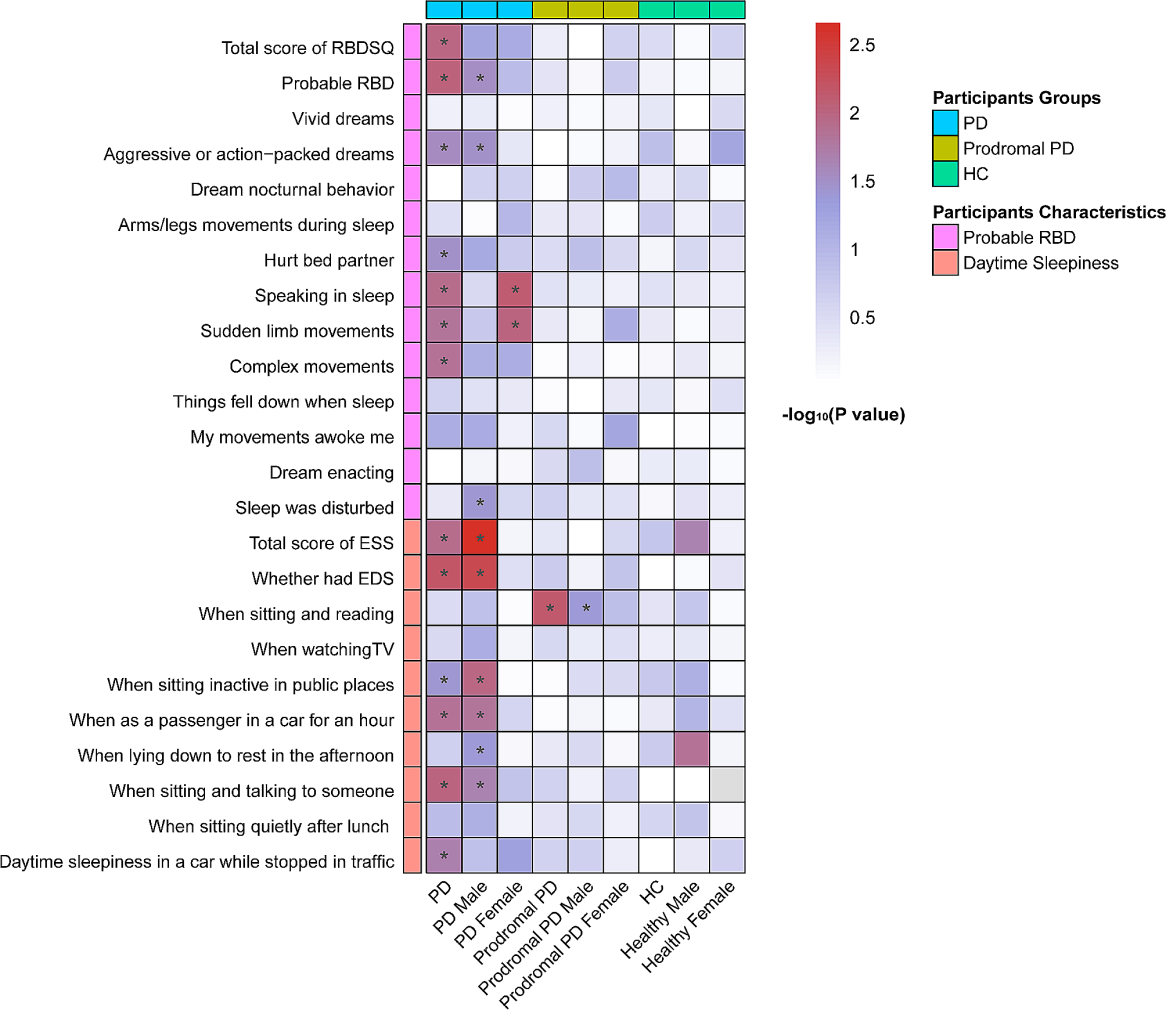
Associations of sleep disorders with serum NfL levels in longitudinal analyses. *P < 0.05

**Fig. 5:**
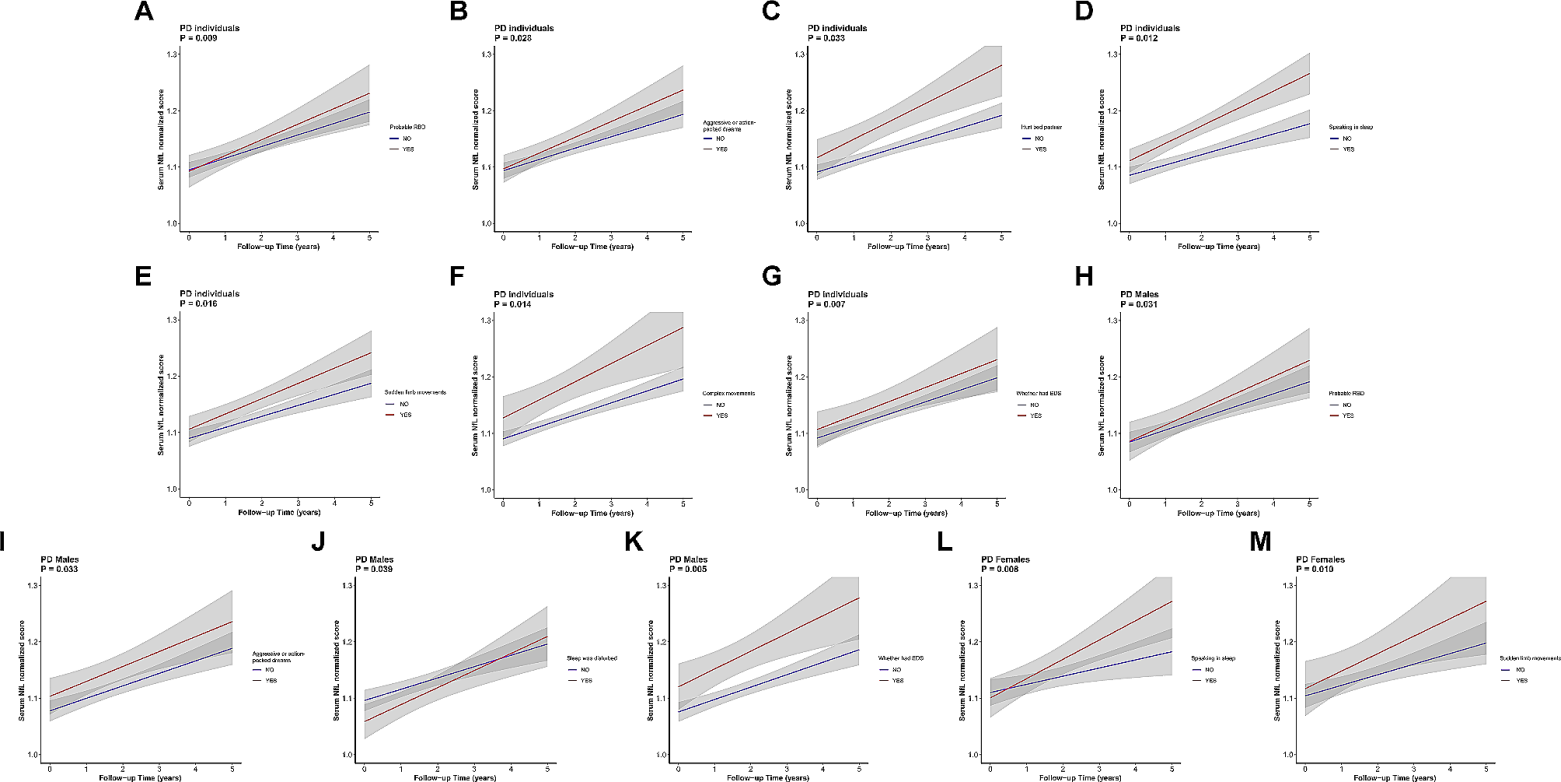
Longitudinal associations of sleep disorders with serum NfL levels PD individuals with pRBD (**A**), aggressive or action-packed dreams (**B**), hurting bed partner (**C**), speaking in sleep (**D**), sudden limb movements (**E**), complex movements (**F**), and whether had EDS (**G**), PD males with pRBD (**H**), aggressive or action-packed dreams (**I**), disturbance of sleep (**J**), and whether had EDS (**K**), PD females with speaking in sleep (**L**) and sudden limb movements (**M**) contribute to increased levels of Serum NfL

After excluding one PD individual without follow-up data on serum NfL, 1112 individuals (PD: *n* = 584; prodromal PD: *n* = 353; HC: *n* = 175) were included for our longitudinal analysis. In addition to baseline measures, these participants had at least one serum NfL measurement over a five-year follow-up period. We found PD participants who had higher RBDSQ scores (*β* = 0.002; *P* = 0.011) (Fig. 4 and Supplementary Table S7) and those who were considered as pRBD (*β* = 0.012; *P* = 0.009) (Fig. 5A) showed an increasing trend of serum NfL levels. Furthermore, specific RBD behaviors, such as aggressive or action-packed dreams (*β* = 0.009; *P* = 0.028) (Fig. 5B), hurting bed partner (*β* = 0.011; *P* = 0.033) (Fig. 5C), speaking in sleep (*β* = 0.010; *P* = 0.012) (Fig. 5D), sudden limb movements (*β* = 0.009; *P* = 0.016) (Fig. 5E), and complex movements (*β* = 0.016; *P* = 0.014) (Fig. 4F) were observably correlated with a longitudinal increase in serum NfL levels among PD patients. Moreover, PD participants who had higher total ESS scores (*β* = 0.001; *P* = 0.012) (Supplementary Table S8) or who were considered as EDS (*β* = 0.013; *P* = 0.007) (Fig. 5G) showed an increasing trend of serum NfL levels. As for ESS items, there were significant associations between daytime sleepiness on four specific situations and an increase in serum NfL levels. These four situations included stopped in traffic in a car (*β* = 0.014; *P* = 0.021), staying in a car as a passenger for an hour without a break (*β* = 0.005; *P* = 0.014), sitting and talking to someone (*β* = 0.017; *P* = 0.010), and sitting inactive in public places (*β* = 0.006; *P* = 0.041).

We further conducted subgroup analyses stratified by gender. In the male subgroup, PD participants who was considered as pRBD (*β* = 0.011; *P* = 0.031) (Fig. 5H) and those who displayed two specific RBD behaviors (aggressive or action-packed dreams (*β* = 0.011; *P* = 0.033) (Fig. 5I), disturbed sleep (*β* = 0.010; *P* = 0.039) (Fig. 5J)) showed an increasing trend of serum NfL levels. In terms of daytime sleepiness, PD males who had a higher score of ESS (*β* = 0.002; *P* = 0.002), those who were considered as EDS (*β* = 0.017; *P* = 0.005) (Fig. 5K), and those had possibilities of daytime sleepiness on four conditions (sitting inactive in public places (*β* = 0.008; *P* = 0.010), as a passenger staying in a car for an hour without a break (*β* = 0.006; *P* = 0.015), lying down to rest in the afternoon (*β* = 0.005; *P* = 0.043), sitting and talking to someone (*β* = 0.017; *P* = 0.023)) also showed an increasing trend of in serum NfL levels. In the female subgroup, only two specific RBD behaviors (speaking in sleep (*β* = 0.017; *P* = 0.008) (Fig. 5L), sudden limb movements (*β* = 0.021; *P* = 0.010) (Fig. 5M)) were associated with an increasing trend of serum NfL levels.

As for prodromal PD individuals, only one ESS item of possibilities of daytime sleepiness when sitting and reading (*β* = 0.009; *P* = 0.007) showed a significant positive association with increased serum NfL levels (Supplementary Table S10). This result was also observed in prodromal PD males (Supplementary Table S10).

In HCs, we identified no association between sleep disorders and serum NfL levels via longitudinal-sectional analyses (Supplementary Table S11 and S12).

### Associations between longitudinal changes in ESS scores and change rates of serum NfL levels

**Fig. 6:**
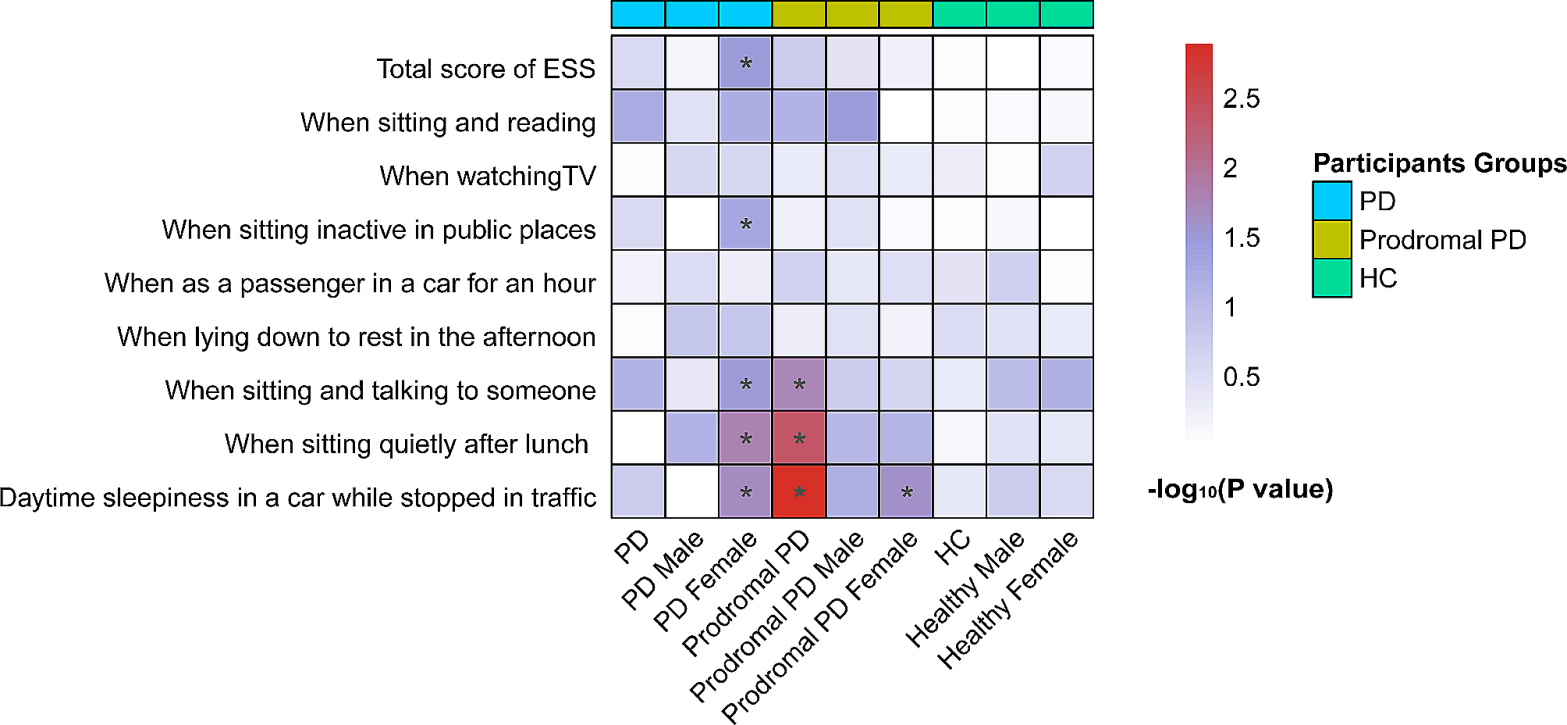
Associations between change rates of daytime sleepiness scores and change rates of serum NfL levels. *P < 0.05

**Fig. 7:**
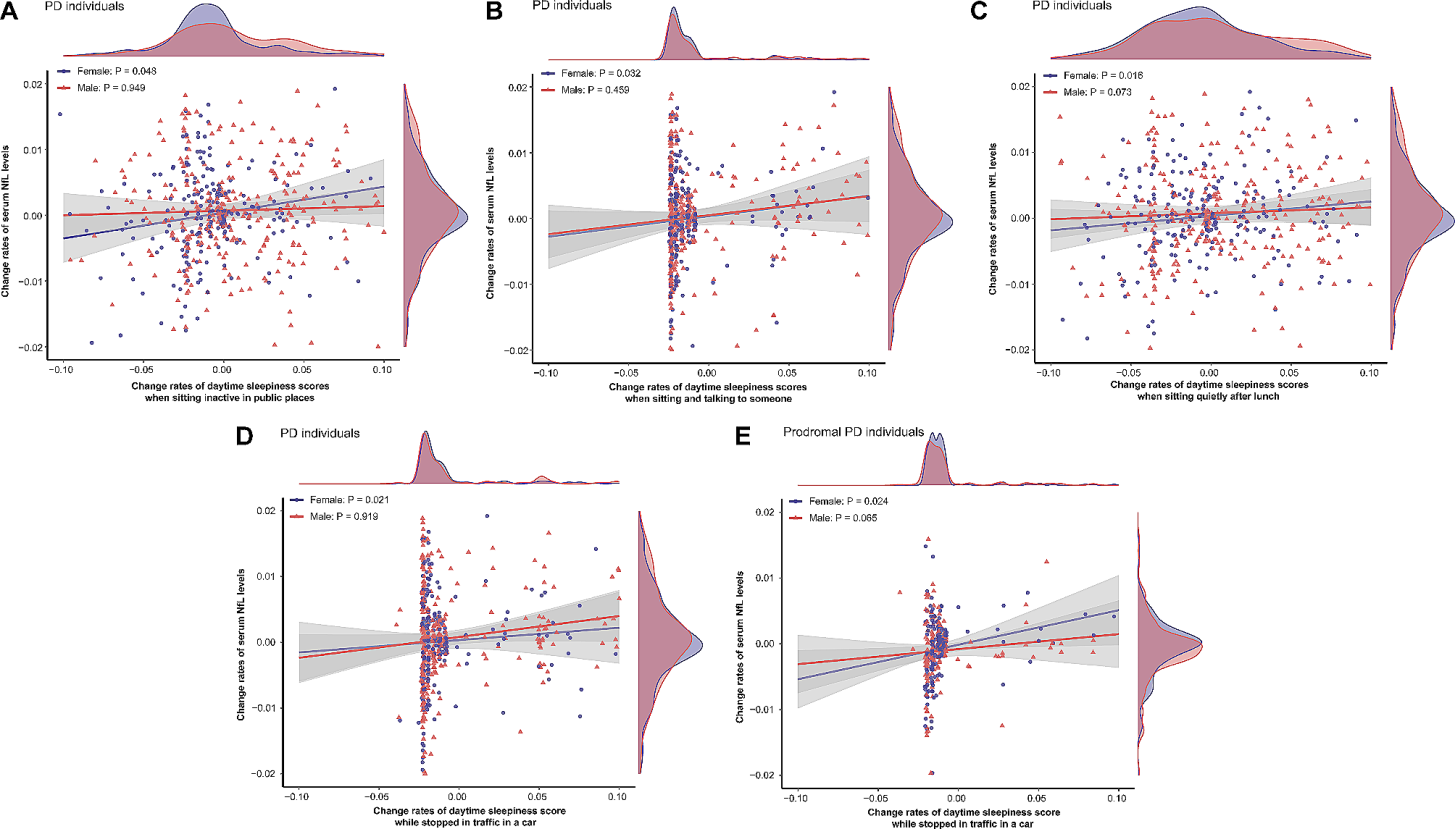
Associations between change rates of daytime sleepiness scores and change rates of serum NfL levels. Increased possibilities of daytime sleepiness when sitting inactive in public places (**A**), sitting and talking to someone (**B**), sitting quietly after lunch (**C**), and stopping in traffic in a car (**D**) in PD females are associated with faster increases of serum NfL levels. In prodromal PD females, increased possibilities of daytime sleepiness while stopped in traffic in a car (**E**), are also associated with faster increases in serum NfL levels

After excluding 15 participants without change rates of ESS scores, our analysis enrolled 1097 individuals (PD: *n* = 581; prodromal PD: *n* = 342; HC: *n* = 174) (Fig. 6). The increased total score of ESS (*β* = 0.004; *P* = 0.033) and increased probabilities of daytime sleepiness in four different situations (sitting inactive in public places (*β* = 0.036; *P* = 0.048) (Fig. 7A), sitting and talking to someone (*β* = 0.031 ; *P* = 0.032) (Fig. 7B), sitting quietly after lunch (*β* = 0.030; *P* = 0.016) (Fig. 7C), stopped in traffic in a car (*β* = 0.033; *P* = 0.021) (Fig. 7D)) had significant correlations with a notable rise increase in serum NfL levels in PD females, whereas no associations were found in total PD participants and PD males (Supplementary Table S13). In prodromal PD participants, increased possibilities of daytime sleepiness on three situations (sitting and talking to someone (*β* = 0.021; *P* = 0.018), sitting quietly after lunch (*β* = 0.021; *P* = 0.004), stopped in traffic in a car (*β* = 0.028; *P* = 0.001)) were significantly correlated with a greater rate of the increase in serum NfL levels (Supplementary Table S14). When stratified by gender, the possibilities of daytime sleepiness only in one situation (stopped in traffic in a car (*β* = 0.039; *P* = 0.024) (Fig. 7E)) was significantly positive correlated with the rate of increase in serum NfL levels in females, while no significant association was revealed in the male subgroup (Supplementary Table S14).

As for HCs, we found no significant association between the possibilities of daytime sleepiness and change rates of serum NfL levels (Supplementary Table S15).

## Discussion

The present study revealed that different RBD and ESS items in three diagnostic groups were strongly correlated with serum NfL levels. Moreover, significant disparities were seen in the baseline levels of serum NfL across the three diagnostic groups, with the PD group having the highest serum NfL levels. The findings of our research indicated that serum NfL levels in PD individuals were influenced by RBD and EDS behaviors, where there were gender differences. In prodromal PD participants, significant associations were seen between some specific RBD behaviors and ESS items and higher serum NfL levels. In HCs, we only discovered a significant correlation between possibilities of daytime sleepiness when sitting more quietly after lunch and more elevated serum NFL levels, which was still significant in the male subgroup. These findings indicated that sleep disorders might be a marker of the severity of neurological damage and PD progression. Good sleep may have neuroprotective effects, thus attention should paid to sleep management for PD prevention.

Clinical studies on the pathogenesis of RBD have suggested that the potential cause of RBD may include the degeneration or dysfunction of the brainstem circuits responsible for regulating REM sleep [20, 21]. Aside from that, previous research has demonstrated that RBD falls under the category of “diffuse malignant phenotype”, which designates a more severe subtype of synaptic neuropathy with more pronounced dopaminergic impairments [22]. Therefore, we can infer that PD patients with the RBD subtype are more severely affected, which is also supported by pathological autopsy studies [23, 24]. Nevertheless, serum NfL has not been adequately studied as a promising biomarker for monitoring the course of the disease and its correlation with RBD. Serum NfL is considered a potentially useful biomarker for the RBD subgroup in a study on the associations of serum NfL and glial fibrillary acidic protein with the RBD subtype of PD, which is consistent with our results [25]. Another study exploring the relationship between plasma NfL levels and cognitive function in PD individuals also observed significantly higher plasma NfL levels in individuals with RBD compared to those without RBD [26]. In contrast, a newly published study on the correlation of non-motor markers and neuronal damage in patients with early Parkinson’s disease found no link between plasma NfL levels and the presence of an RBD [27]. This discrepancy might result from the bias in the assessment of sleep traits and variations in sample sizes.

NfL can serve as a marker of axonal injury because it is stable in axons under physiologic circumstances [28]. In contrast, following axonal damage, neurofilaments diffuse into the CSF and then they are discharged into blood circulation through the arachnoid villi [29, 30]. Significantly, a longitudinal study involving 40 individuals with chronic insomnia disorder (CID) revealed a correlation between heightened levels of serum NfL (indicating functional and structural damage to neurons, axons, and glial cells) and both subjective and objective sleep parameters among CID individuals [31]. A possible mechanism linking sleep disorders with neuronal damage has been proposed by previous studies. Since a good night’s sleep can enhance the clearance of potentially neurotoxic waste products from the glymphatic system that accumulate during wakefulness, sleep disorders can increase the production of reactive oxygen species (a kind of toxic metabolites) which damage neurons [32]. Therefore, as a type of sleep disorders, RBD may affect cerebrospinal fluid flow and promote glymphatic system dysfunction, which might inhibit the clearance of metabolic waste and eventually cause neuronal damage [33].

There are several potential mechanisms underlying the associations between EDS-related brain structural alterations and neurodegeneration. Firstly, PD progression may be accompanied by degeneration of the neurons that affect wakefulness and sleep, which raises the possibility of sleep disorders (including EDS) in PD patients. Previous studies have found that CSF production correlates with changes in circadian rhythms, and human CSF production peaks during sleep after midnight, as measured by magnetic resonance imaging [34, 35]. EDS is one of the common symptoms of circadian rhythm disturbance and it may predate Lewy pathology (LP), a marker of PD pathogenesis [36]. The relationship between EDS and widespread topographic LP expansion might further support the early finding of an association between EDS and PD [37]. Since the clock gene is central to circadian rhythms, its altered expression in PD disrupts the 24-hour cycle, leading to impaired nighttime sleep and further impaired function of the lymphatic system [38].

Although the mechanisms underlying the association of EDS with elevated serum NfL levels have not been elucidated, currently there are two reasons to account for the association. Firstly, evidence showed that EDS might be an indication of inadequate clearance of neurotoxic metabolic by-products during sleep or neurodegeneration in areas associated with the maintenance of the wake state [33]. In addition, a previous study has shown that as people with EDS get older or enter late middle age, their cortical thickness decreases [39]. A community-based longitudinal study (the MAP cohort) found that lower inferior lateral orbitofrontal cortex and inferior frontal orbital gyrus grey matter volumes were associated with greater sleep fragmentation (including RBD) in older community adults [40]. And a short-term follow-up study also found that increased plasma NfL was associated with cortical thinning [41]. Therefore, we speculate that EDS is associated with increased serum NfL levels, possibly via EDS-related brain structural alterations. This is consistent with previous findings on the correlation between EDS and CSF biomarkers of inflammation and axonal integrity in cognitively unimpaired older adults [39]. The correlation between EDS and NfL in HC group can thus be explained. Secondly, EDS is thought to be a consequence of OSA [22, 42]. OSA induces cerebral hypoperfusion, leading to increased oxidative stress and subsequently neuronal and axonal damage [43]. Upon axonal damage, the release of NfL occurred first into CSF and then into the blood, leading to increased levels of NfL in the serum [44].

The association between sleep disorders and serum NfL levels was investigated using a cross-sectional and prospective follow-up study design in an effort to improve the study’s scientific validity. Moreover, unlike CSF, which must be collected via a problematic process that frequently involves a puncture, serum biomarkers are simple to acquire.

This study represents the first comprehensive exploration of the association between sleep disorders and serum NfL levels in individuals experiencing early and prodromal PD, thus addressing a notable research gap in this domain. Moreover, the cross-sectional and prospective follow-up design employed in this investigation distinctly bolsters the scientific validity and effectiveness of our findings. Our study offers a more nuanced depiction of the relationship between sleep disorders and serum NfL levels. Compared with biomarkers that require complex procedures such as cerebrospinal fluid puncture for collection, serum samples are simple to obtain, non-invasive, and easily applicable in a wide range of clinical and research settings. Despite these advantages, the study faced some limitations that warrant consideration in future studies. Firstly, assessments such as RBDSQ and ESS rely on self-reported sleep questionnaires, which are subject to various subjective factors. Therefore, future studies should incorporate objective indicators such as nocturnal polysomnography and pathological evidence. Secondly, potential missing data in our follow-up assessments may affect the reliability of our results. Thus, a wider cohort study is needed in the future with measures to minimize missing data and ensure the stability and credibility of the results. Lastly, it is important to note that our blood samples were stored at -80℃ for a period, and previous related studies have suggested that this may lead to alterations in concentration [45]. Future studies should control for this variable. Finally, the sample size of participants with both EDS and pRBD was small in this study. Therefore, it is necessary to validate our findings in a larger sample size in order to enhance the generalisability and applicability of the findings.

## Conclusion

Sleep disorders are significantly associated with high levels of serum NfL in the prodromal and early stages of PD. This finding underscores the potential of sleep disorders as a robust clinical indicator of neuronal degeneration, closely linked with PD progression. Consequently, timely interventions and therapeutic measures assume paramount importance in PD management, particularly during the prodromal and early phases of the disease. The timely identification and intervention of sleep disorders hold promise in not only decelerating the neurodegenerative processes associated with PD but also substantially enhancing patients’ quality of life. Furthermore, routine monitoring of NfL levels emerges as an effective and dynamic approach for tracking sleep disorders and PD progression, opening up new avenues for enhancing overall health and quality of life in PD patients.

### Electronic supplementary material

Below is the link to the electronic supplementary material.

Supplementary Material 1

## Data Availability

The data supporting the findings of this study are openly available in PPMI datebase at (http://www.ppmi-info.org/data). The datasets used and/or analysed during the current study are available from the corresponding author on reasonable request.

## Abbreviations

PD: Parkinson’s disease
NfL: neurofilament light chain
HCs: healthy controls
RBD: rapid eye movement sleep behavior disorder
EDS: excessive daytime sleepiness
NMS: non-motor symptoms
RLS: restless legs syndrome
OSA: obstructive sleep apnea
REM: rapid eye movement
CSF: cerebrospinal fluid
APD: atypical parkinsonian disorders
PPMI: Parkinson’s Progression Markers Initiative
LONI: Laboratory of NeuroImaging
RBDSQ: Rapid Eye Movement Sleep Behavioural Disorder Screening Questionnaire
ESS: Epworth Sleepiness Scale
LRRK2: Leucine-rich repeat kinase 2
GBA: Glucosylceramidaseβ
SNCA: Alpha-Synuclein
MoCA: Montreal Cognitive Assessment
CID: chronic insomnia disorder
LP: Lewy pathology
