# Supplementary material for: Associations of sleep disorders with serum neurofilament light chain levels in Parkinson’s disease
Source: BMC Neurol. 2024 May 1;24:147. doi: 10.1186/s12883-024-03642-y (PMC11061948; doi:10.1186/s12883-024-03642-y)
Supplement: Supplementary file 1 — Supplementary Material 1 [file 12883_2024_3642_MOESM1_ESM.docx]

**Title: Associations of sleep disorders with serum neurofilament light chain levels in Parkinson’s disease**

**CONTENT**

| **TITLE** |  | **PAGE** |
| --- | --- | --- |
| **Table S1** | Associations of RBD with serum NfL levels in PD individuals in cross-sectional study | 2 |
| **Table S2** | Associations of daytime sleepiness with serum NfL levels in PD individuals in cross-sectional study | 3 |
| **Table S3** | Associations of RBD with serum NfL levels in Prodromal PD individuals in cross-sectional study | 4 |
| **Table S4** | Associations of daytime sleepiness with serum NfL levels in Prodromal PD individuals in cross-sectional study | 5 |
| **Table S5** | Associations of RBD with serum NfL levels in HCs in cross-sectional study | 6 |
| **Table S6** | Associations of daytime sleepiness with serum NfL levels in HCs in cross-sectional study | 7 |
| **Table S7** | Associations of RBD with serum NfL levels in PD individuals in longitudinal study | 8 |
| **Table S8** | Associations of daytime sleepiness with serum NfL levels in PD individuals in longitudinal study | 9 |
| **Table S9** | Associations of RBD with serum NfL levels in Prodromal PD individuals in longitudinal study | 10 |
| **Table S10** | Associations of daytime sleepiness with serum NfL levels in Prodromal PD individuals in longitudinal study | 11 |
| **Table S11** | Associations of RBD with serum NfL levels in HCs in longitudinal study | 12 |
| **Table S12** | Associations of daytime sleepiness with serum NfL levels in HCs in longitudinal study | 13 |
| **Table S13** | Associations between change rates of daytime sleepiness and changes of serum NfL in PD individuals | 14 |
| **Table S14** | Associations between change rates of daytime sleepiness and changes of serum NfL in prodromal PD individuals | 15 |
| **Table S15** | Associations between change rates of daytime sleepiness and changes of serum NfL in HCs | 16 |

| **Table S1. Associations of RBD with serum NfL levels in PD individuals in cross-sectional study** | | | | | | | | |
| --- | --- | --- | --- | --- | --- | --- | --- | --- |
| **Sleep characteristics** | **PD** | |  | **PD Male** | |  | **PD Female** | |
|  | **β** | **P^†^** |  | **β** | **P^†^** |  | **β** | **P^†^** |
| Total score of RBDSQ | 0.004 | 0.059 |  | 0.003 | 0.229 |  | 0.006 | 0.118 |
| Probable RBD (total score of RBDSQ≥6) | 0.014 | 0.386 |  | 0.004 | 0.857 |  | 0.034 | 0.224 |
| Vivid Dreams | <0.001 | 0.996 |  | 0.003 | 0.872 |  | -0.002 | 0.908 |
| Aggressive or Action-packed dreams | 0.023 | 0.107 |  | 0.023 | 0.206 |  | 0.025 | 0.293 |
| Dream nocturnal behavior | 0.033 | **0.042** |  | 0.016 | 0.441 |  | 0.054 | **0.032** |
| Move arms/legs during sleep | 0.027 | **0.049** |  | 0.017 | 0.294 |  | 0.043 | 0.067 |
| Hurt bed partner | 0.021 | 0.280 |  | 0.007 | 0.724 |  | 0.075 | 0.084 |
| Speaking in sleep | 0.018 | 0.180 |  | 0.029 | 0.103 |  | 0.003 | 0.906 |
| Sudden limb movements | 0.010 | 0.462 |  | 0.006 | 0.712 |  | 0.019 | 0.448 |
| Complex movements | 0.035 | 0.097 |  | 0.035 | 0.186 |  | 0.036 | 0.330 |
| Things fell down when sleep | 0.050 | 0.063 |  | 0.100 | **0.004** |  | -0.022 | 0.603 |
| My movements awake me | 0.011 | 0.459 |  | 0.019 | 0.302 |  | -0.003 | 0.917 |
| Dream enacting | 0.004 | 0.766 |  | -0.014 | 0.427 |  | 0.027 | 0.198 |
| Sleep is disturbed | -0.001 | 0.930 |  | -0.010 | 0.588 |  | 0.014 | 0.532 |

PD: Parkinson’s disease; NfL: Neurofilament light chain; RBD: Rapid eye movement sleep Behavior Disorder; RBDSQ: RBD Screening Questionnaire

†: adjusted for age, sex, years of education and disease duration; Bold text: significant results

| **Table S2. Associations of daytime sleepiness with serum NfL levels in PD individuals in cross-sectional study** | | | | | | | | |
| --- | --- | --- | --- | --- | --- | --- | --- | --- |
| **Sleep characteristics** | **PD** | |  | **PD Male** | |  | **PD Female** | |
|  | **β** | **P^†^** |  | **β** | **P^†^** |  | **β** | **P^†^** |
| Total score of ESS | <-0.001 | 0.806 |  | <-0.001 | 0.861 |  | -0.001 | 0.823 |
| Whether had EDS (total score of ESS ≥ 10) | -0.007 | 0.685 |  | -0.014 | 0.530 |  | 0.004 | 0.888 |
| Possibilities of daytime sleepiness when sitting and reading | -0.008 | 0.237 |  | -0.008 | 0.412 |  | -0.009 | 0.395 |
| Possibilities of daytime sleepiness when watching TV | 0.008 | 0.289 |  | 0.005 | 0.606 |  | 0.011 | 0.328 |
| Possibilities of daytime sleepiness when sitting, inactive in a public place | -0.005 | 0.608 |  | -0.008 | 0.511 |  | -0.002 | 0.904 |
| Possibilities of daytime sleepiness when one as a passenger stays in a car for an hour without a break | -0.002 | 0.731 |  | -0.009 | 0.312 |  | 0.006 | 0.584 |
| Possibilities of daytime sleepiness when lying down to rest in the afternoon | -0.004 | 0.530 |  | 0.002 | 0.806 |  | -0.012 | 0.229 |
| Possibilities of daytime sleepiness when sitting and talking to someone | 0.031 | 0.125 |  | 0.034 | 0.153 |  | 0.022 | 0.548 |
| Possibilities of daytime sleepiness when sitting quietly after lunch | -0.003 | 0.731 |  | 0.001 | 0.959 |  | -0.008 | 0.521 |
| Possibilities of daytime sleepiness in a car, while stopped in traffic | 0.013 | 0.473 |  | 0.014 | 0.573 |  | 0.011 | 0.683 |

PD: Parkinson’s disease; NfL: Neurofilament light chain; ESS: Epworth Sleepiness Scale; EDS: excessive daytime sleepiness

†: adjusted for age, sex, years of education and and disease duration;

| **Table S3. Associations of RBD with serum NfL levels in Prodromal PD in cross-sectional study** | | | | | | | | |
| --- | --- | --- | --- | --- | --- | --- | --- | --- |
| **Sleep characteristics** | **Prodromal PD** | |  | **Prodromal PD Male** | |  | **Prodromal PD Female** | |
|  | **β** | **P^†^** |  | **β** | **P^†^** |  | **β** | **P^†^** |
| Total score of RBDSQ | 0.005 | 0.154 |  | 0.008 | 0.100 |  | 0.002 | 0.738 |
| Probable RBD (total score of RBDSQ≥6) | -0.007 | 0.774 |  | 0.032 | 0.356 |  | -0.052 | 0.166 |
| Vivid Dreams | 0.016 | 0.363 |  | 0.052 | **0.048** |  | -0.014 | 0.584 |
| Aggressive or Action-packed dreams | 0.004 | 0.839 |  | 0.013 | 0.642 |  | -0.011 | 0.713 |
| Dream nocturnal behavior | 0.025 | 0.220 |  | 0.016 | 0.587 |  | 0.033 | 0.255 |
| Move arms/legs during sleep | 0.027 | 0.153 |  | 0.035 | 0.188 |  | 0.022 | 0.433 |
| Hurt bed partner | 0.031 | 0.381 |  | 0.037 | 0.375 |  | 0.018 | 0.773 |
| Speaking in sleep | -0.006 | 0.739 |  | -0.013 | 0.681 |  | -0.002 | 0.928 |
| Sudden limb movements | -0.004 | 0.838 |  | 0.017 | 0.548 |  | -0.030 | 0.311 |
| Complex movements | 0.013 | 0.647 |  | 0.005 | 0.895 |  | 0.026 | 0.565 |
| Things fell down when sleep | -0.002 | 0.951 |  | 0.004 | 0.929 |  | -0.011 | 0.862 |
| My movements awake me | 0.018 | 0.432 |  | 0.018 | 0.572 |  | 0.018 | 0.611 |
| Dream enacting | 0.009 | 0.601 |  | 0.026 | 0.303 |  | -0.005 | 0.824 |
| Sleep is disturbed | 0.038 | **0.026** |  | 0.044 | 0.076 |  | 0.029 | 0.233 |

PD: Parkinson’s disease; NfL: Neurofilament light chain; RBD: Rapid eye movement sleep Behavior Disorder; RBDSQ: RBD Screening Questionnaire

†: adjusted for age, sex, years of education and diagnoses (RBD, hyposmia and and genetic mutation); Bold text: significant results

| **Table S4. Associations of daytime sleepiness with serum NfL levels in Prodromal PD individuals in cross-sectional study** | | | | | | | | |
| --- | --- | --- | --- | --- | --- | --- | --- | --- |
| **Sleep characteristics** | **Prodromal PD** | |  | **Prodromal PD Male** | |  | **Prodromal PD Female** | |
|  | **β** | **P^†^** |  | **β** | **P^†^** |  | **β** | **P^†^** |
| Total score of ESS | -0.001 | 0.673 |  | 0.001 | 0.734 |  | -0.005 | 0.209 |
| Whether had EDS (total score of ESS ≥ 10) | 0.020 | 0.438 |  | 0.033 | 0.335 |  | -0.011 | 0.796 |
| Possibilities of daytime sleepiness when sitting and reading | -0.004 | 0.665 |  | 0.012 | 0.425 |  | -0.020 | 0.141 |
| Possibilities of daytime sleepiness when watching TV | -0.009 | 0.352 |  | -0.008 | 0.579 |  | -0.013 | 0.333 |
| Possibilities of daytime sleepiness when sitting, inactive in a public place | -0.013 | 0.325 |  | -0.016 | 0.393 |  | -0.014 | 0.473 |
| Possibilities of daytime sleepiness when one as a passenger stays in a car for an hour without a break | 0.005 | 0.620 |  | 0.016 | 0.276 |  | -0.007 | 0.595 |
| Possibilities of daytime sleepiness when lying down to rest in the afternoon | -0.001 | 0.864 |  | 0.001 | 0.926 |  | -0.007 | 0.586 |
| Possibilities of daytime sleepiness when sitting and talking to someone | 0.005 | 0.882 |  | -0.004 | 0.926 |  | 0.012 | 0.829 |
| Possibilities of daytime sleepiness when sitting quietly after lunch | 0.002 | 0.898 |  | 0.013 | 0.406 |  | -0.024 | 0.262 |
| Possibilities of daytime sleepiness in a car, while stopped in traffic | -0.004 | 0.886 |  | 0.008 | 0.787 |  | 0.009 | 0.905 |

PD: Parkinson’s disease; NfL: Neurofilament light chain; ESS: Epworth Sleepiness Scale; EDS: excessive daytime sleepiness

†: adjusted for age, sex, years of education and diagnoses (RBD, hyposmia and and genetic mutation);

| **Table S5. Associations of RBD with serum NfL levels in HCs in cross-sectional study** | | | | | | | | |
| --- | --- | --- | --- | --- | --- | --- | --- | --- |
| **Sleep characteristics** | **HC** | |  | **Healthy Male** | |  | **Healthy Female** | |
|  | **β** | **P^†^** |  | **β** | **P^†^** |  | **β** | **P^†^** |
| Total score of RBDSQ | 0.003 | 0.603 |  | 0.009 | 0.171 |  | -0.009 | 0.346 |
| Probable RBD (total score of RBDSQ≥6) | -0.007 | 0.849 |  | -0.002 | 0.962 |  | -0.011 | 0.867 |
| Vivid Dreams | -0.002 | 0.945 |  | 0.035 | 0.240 |  | -0.072 | 0.098 |
| Aggressive or Action-packed dreams | -0.015 | 0.624 |  | 0.006 | 0.864 |  | -0.072 | 0.212 |
| Dream nocturnal behavior | 0.018 | 0.537 |  | 0.051 | 0.160 |  | -0.056 | 0.301 |
| Move arms/legs during sleep | 0.011 | 0.684 |  | 0.028 | 0.386 |  | -0.034 | 0.507 |
| Hurt bed partner | -0.011 | 0.854 |  | 0.070 | 0.305 |  | -0.213 | 0.074 |
| Speaking in sleep | -0.008 | 0.774 |  | 0.019 | 0.574 |  | -0.056 | 0.233 |
| Sudden limb movements | 0.004 | 0.904 |  | 0.024 | 0.527 |  | -0.027 | 0.665 |
| Complex movements | 0.057 | 0.381 |  | 0.050 | 0.574 |  | 0.087 | 0.380 |
| Things fell down when sleep | 0.026 | 0.675 |  | 0.046 | 0.670 |  | 0.013 | 0.872 |
| My movements awake me | 0.048 | 0.191 |  | 0.047 | 0.262 |  | 0.050 | 0.486 |
| Dream enacting | 0.015 | 0.529 |  | 0.016 | 0.569 |  | 0.000 | 0.996 |
| Sleep is disturbed | 0.003 | 0.905 |  | -0.022 | 0.532 |  | 0.048 | 0.328 |

HC: Healthy Control; NfL: Neurofilament light chain; RBD: Rapid eye movement sleep Behavior Disorder; RBDSQ: RBD Screening Questionnaire

†: adjusted for age, sex,and years of education;

| **Table S6. Associations of daytime sleepiness with serum NfL levels in HCs in cross-sectional study** | | | | | | | | |
| --- | --- | --- | --- | --- | --- | --- | --- | --- |
| **Sleep characteristics** | **HC** | |  | **Healthy Male** | |  | **Healthy Female** | |
|  | **β** | **P^†^** |  | **β** | **P^†^** |  | **β** | **P^†^** |
| Total score of ESS | -0.001 | 0.846 |  | 0.003 | 0.500 |  | -0.009 | 0.184 |
| Whether had EDS (total score of ESS ≥ 10) | 0.034 | 0.359 |  | 0.035 | 0.375 |  | 0.036 | 0.688 |
| Possibilities of daytime sleepiness when sitting and reading | -0.010 | 0.543 |  | -0.004 | 0.828 |  | -0.015 | 0.597 |
| Possibilities of daytime sleepiness when watching TV | -0.012 | 0.426 |  | -0.008 | 0.677 |  | -0.021 | 0.419 |
| Possibilities of daytime sleepiness when sitting, inactive in a public place | -0.017 | 0.316 |  | -0.016 | 0.398 |  | -0.032 | 0.428 |
| Possibilities of daytime sleepiness when one as a passenger stays in a car for an hour without a break | -0.009 | 0.503 |  | 0.014 | 0.381 |  | -0.052 | 0.034 |
| Possibilities of daytime sleepiness when lying down to rest in the afternoon | -0.002 | 0.867 |  | 0.010 | 0.508 |  | -0.022 | 0.274 |
| Possibilities of daytime sleepiness when sitting and talking to someone | 0.034 | 0.713 |  | 0.039 | 0.660 |  | - | - |
| Possibilities of daytime sleepiness when sitting quietly after lunch | 0.046 | **0.007** |  | 0.046 | **0.011** |  | 0.049 | 0.272 |
| Possibilities of daytime sleepiness in a car, while stopped in traffic | -0.004 | 0.901 |  | 0.006 | 0.861 |  | -0.089 | 0.462 |

HC: Healthy Control; NfL: Neurofilament light chain; ESS: Epworth Sleepiness Scale; EDS: excessive daytime sleepiness

†: adjusted for age, sex and years of education; Bold text: significant results

| **Table S7. Associations of RBD with serum NfL levels in PD individuals in longitudinal study** | | | | | | | | |
| --- | --- | --- | --- | --- | --- | --- | --- | --- |
| **Sleep characteristics** | **PD** | |  | **PD Male** | |  | **PD Female** | |
|  | **β** | **P^†^** |  | **β** | **P^†^** |  | **β** | **P^†^** |
| Total score of RBDSQ | 0.002 | **0.011** |  | 0.002 | 0.061 |  | 0.002 | 0.071 |
| Probable RBD (total score of RBDSQ≥6) | 0.012 | **0.009** |  | 0.011 | **0.031** |  | 0.014 | 0.125 |
| Vivid Dreams | 0.002 | 0.607 |  | 0.003 | 0.541 |  | 0.001 | 0.901 |
| Aggressive or Action-packed dreams | 0.009 | **0.028** |  | 0.011 | **0.033** |  | 0.006 | 0.447 |
| Dream nocturnal behavior | <0.001 | 0.994 |  | -0.007 | 0.235 |  | 0.009 | 0.228 |
| Move arms/legs during sleep | 0.004 | 0.355 |  | <0.001 | 0.914 |  | 0.012 | 0.105 |
| Hurt bed partner | 0.011 | **0.033** |  | 0.010 | 0.069 |  | 0.018 | 0.189 |
| Speaking in sleep | 0.010 | **0.012** |  | 0.005 | 0.312 |  | 0.017 | **0.008** |
| Sudden limb movements | 0.009 | **0.016** |  | 0.006 | 0.177 |  | 0.021 | **0.010** |
| Complex movements | 0.016 | **0.014** |  | 0.013 | 0.084 |  | 0.020 | 0.075 |
| Things fell down when sleep | 0.011 | 0.246 |  | 0.010 | 0.377 |  | 0.011 | 0.467 |
| My movements awake me | 0.008 | 0.076 |  | 0.009 | 0.070 |  | 0.004 | 0.611 |
| Dream enacting | <0.001 | 0.942 |  | 0.002 | 0.708 |  | -0.002 | 0.769 |
| Sleep is disturbed | 0.003 | 0.470 |  | 0.010 | **0.039** |  | -0.007 | 0.273 |

PD: Parkinson’s disease; NfL: Neurofilament light chain; RBD: Rapid eye movement sleep Behavior Disorder; RBDSQ: RBD Screening Questionnaire

†: adjusted for age, sex, years of education and and disease duration; Bold text: significant results

| **Table S8. Associations of daytime sleepiness with serum NfL levels in PD individuals in longitudinal study** | | | | | | | | |
| --- | --- | --- | --- | --- | --- | --- | --- | --- |
| **Sleep characteristics** | **PD** | |  | **PD Male** | |  | **PD Female** | |
|  | **β** | **P^†^** |  | **β** | **P^†^** |  | **β** | **P^†^** |
| Total score of ESS | 0.001 | **0.012** |  | 0.002 | **0.002** |  | <0.001 | 0.681 |
| Whether had EDS (total score of ESS ≥ 10) | 0.013 | **0.007** |  | 0.017 | **0.005** |  | 0.008 | 0.352 |
| Possibilities of daytime sleepiness when sitting and reading | 0.002 | 0.327 |  | 0.004 | 0.138 |  | <-0.001 | 0.889 |
| Possibilities of daytime sleepiness when watching TV | 0.002 | 0.298 |  | 0.004 | 0.079 |  | -0.001 | 0.682 |
| Possibilities of daytime sleepiness when sitting, inactive in a public place | 0.006 | **0.041** |  | 0.008 | **0.010** |  | 0.001 | 0.859 |
| Possibilities of daytime sleepiness when one as a passenger stays in a car for an hour without a break | 0.005 | **0.014** |  | 0.006 | **0.015** |  | 0.004 | 0.273 |
| Possibilities of daytime sleepiness when lying down to rest in the afternoon | 0.002 | 0.222 |  | 0.005 | **0.043** |  | -0.001 | 0.750 |
| Possibilities of daytime sleepiness when sitting and talking to someone | 0.017 | **0.010** |  | 0.017 | **0.023** |  | 0.021 | 0.150 |
| Possibilities of daytime sleepiness when sitting quietly after lunch | 0.004 | 0.120 |  | 0.005 | 0.083 |  | 0.002 | 0.648 |
| Possibilities of daytime sleepiness in a car, while stopped in traffic | 0.014 | **0.021** |  | 0.011 | 0.140 |  | 0.022 | 0.052 |

PD: Parkinson’s disease; NfL: Neurofilament light chain; ESS: Epworth Sleepiness Scale; EDS: excessive daytime sleepiness

†: adjusted for age, sex, years of education and and disease duration; Bold text: significant results

| **Table S9. Associations of RBD with serum NfL levels in Prodromal PD individuals in longitudinal study** | | | | | | | | |
| --- | --- | --- | --- | --- | --- | --- | --- | --- |
| **Sleep characteristics** | **Prodromal PD** | |  | **Prodromal PD Male** | |  | **Prodromal PD Female** | |
|  | **β** | **P^†^** |  | **β** | **P^†^** |  | **β** | **P^†^** |
| Total score of RBDSQ | <0.001 | 0.572 |  | <-0.001 | 0.963 |  | 0.002 | 0.249 |
| Probable RBD (total score of RBDSQ≥6) | 0.005 | 0.423 |  | 0.002 | 0.788 |  | 0.016 | 0.197 |
| Vivid Dreams | 0.003 | 0.601 |  | 0.002 | 0.843 |  | 0.004 | 0.672 |
| Aggressive or Action-packed dreams | 0.000 | 0.959 |  | -0.002 | 0.844 |  | 0.005 | 0.656 |
| Dream nocturnal behavior | 0.001 | 0.911 |  | -0.011 | 0.200 |  | 0.017 | 0.117 |
| Move arms/legs during sleep | -0.004 | 0.485 |  | -0.007 | 0.407 |  | 0.003 | 0.806 |
| Hurt bed partner | -0.007 | 0.330 |  | -0.013 | 0.131 |  | 0.016 | 0.316 |
| Speaking in sleep | 0.005 | 0.392 |  | 0.005 | 0.514 |  | 0.005 | 0.621 |
| Sudden limb movements | 0.004 | 0.500 |  | -0.003 | 0.732 |  | 0.019 | 0.077 |
| Complex movements | 0.001 | 0.871 |  | 0.005 | 0.565 |  | -0.002 | 0.865 |
| Things fell down when sleep | 0.001 | 0.894 |  | <-0.001 | 0.990 |  | 0.011 | 0.490 |
| My movements awake me | 0.008 | 0.275 |  | 0.002 | 0.822 |  | 0.023 | 0.060 |
| Dream enacting | 0.006 | 0.304 |  | 0.013 | 0.128 |  | -0.003 | 0.783 |
| Sleep is disturbed | 0.007 | 0.233 |  | 0.006 | 0.450 |  | 0.008 | 0.389 |

PD: Parkinson’s disease; NfL: Neurofilament light chain; RBD: Rapid eye movement sleep Behavior Disorder; RBDSQ: RBD Screening Questionnaire

†: adjusted for age, sex, years of education and diagnoses (RBD, hyposmia and and genetic mutation);

| **Table S10. Associations of daytime sleepiness with serum NfL levels in Prodromal PD individuals in longitudinal study** | | | | | | | | |
| --- | --- | --- | --- | --- | --- | --- | --- | --- |
| **Sleep characteristics** | **Prodromal PD** | |  | **Prodromal PD Male** | |  | **Prodromal PD Female** | |
|  | **β** | **P^†^** |  | **β** | **P^†^** |  | **β** | **P^†^** |
| Total score of ESS | 0.001 | 0.434 |  | <-0.000 | 0.973 |  | 0.001 | 0.279 |
| Whether had EDS (total score of ESS ≥ 10) | 0.011 | 0.190 |  | 0.005 | 0.640 |  | 0.020 | 0.160 |
| Possibilities of daytime sleepiness when sitting and reading | 0.009 | **0.007** |  | 0.009 | **0.044** |  | 0.008 | 0.131 |
| Possibilities of daytime sleepiness when watching TV | 0.004 | 0.281 |  | 0.003 | 0.516 |  | 0.005 | 0.363 |
| Possibilities of daytime sleepiness when sitting, inactive in a public place | <0.001 | 0.917 |  | -0.006 | 0.340 |  | 0.007 | 0.303 |
| Possibilities of daytime sleepiness when one as a passenger stays in a car for an hour without a break | 0.001 | 0.876 |  | -0.002 | 0.692 |  | 0.001 | 0.809 |
| Possibilities of daytime sleepiness when lying down to rest in the afternoon | -0.002 | 0.467 |  | -0.004 | 0.310 |  | 0.001 | 0.779 |
| Possibilities of daytime sleepiness when sitting and talking to someone | 0.011 | 0.251 |  | 0.007 | 0.608 |  | 0.020 | 0.239 |
| Possibilities of daytime sleepiness when sitting quietly after lunch | -0.003 | 0.419 |  | -0.004 | 0.280 |  | 0.004 | 0.594 |
| Possibilities of daytime sleepiness in a car, while stopped in traffic | 0.013 | 0.252 |  | 0.014 | 0.220 |  | -0.031 | 0.548 |

PD: Parkinson’s disease; NfL: Neurofilament light chain; ESS: Epworth Sleepiness Scale; EDS: excessive daytime sleepiness

†: adjusted for age, sex, years of education and diagnoses (RBD, hyposmia and and genetic mutation); Bold text: significant results

| **Table S11. Associations of RBD with NfL levels in HCs in longitudinal study** | | | | | | | | |
| --- | --- | --- | --- | --- | --- | --- | --- | --- |
| **Sleep characteristics** | **HC** | |  | **Healthy Male** | |  | **Healthy Female** | |
|  | **β** | **P^†^** |  | **β** | **P^†^** |  | **β** | **P^†^** |
| Total score of RBDSQ | 0.001 | 0.319 |  | <0.001 | 0.822 |  | 0.002 | 0.252 |
| Probable RBD (total score of RBDSQ≥6) | 0.003 | 0.674 |  | 0.002 | 0.809 |  | 0.004 | 0.682 |
| Vivid Dreams | 0.003 | 0.451 |  | <-0.001 | 0.944 |  | 0.007 | 0.297 |
| Aggressive or Action-packed dreams | 0.008 | 0.133 |  | 0.002 | 0.778 |  | 0.018 | 0.060 |
| Dream nocturnal behavior | 0.003 | 0.576 |  | 0.007 | 0.290 |  | -0.002 | 0.809 |
| Move arms/legs during sleep | 0.006 | 0.215 |  | 0.003 | 0.588 |  | 0.009 | 0.261 |
| Hurt bed partner | -0.004 | 0.689 |  | -0.013 | 0.282 |  | 0.015 | 0.421 |
| Speaking in sleep | 0.004 | 0.381 |  | 0.004 | 0.480 |  | 0.004 | 0.565 |
| Sudden limb movements | 0.004 | 0.490 |  | 0.002 | 0.819 |  | 0.007 | 0.483 |
| Complex movements | -0.004 | 0.744 |  | -0.010 | 0.481 |  | 0.006 | 0.712 |
| Things fell down when sleep | 0.009 | 0.442 |  | 0.007 | 0.763 |  | 0.013 | 0.353 |
| My movements awake me | 0.001 | 0.923 |  | 0.001 | 0.895 |  | -0.003 | 0.828 |
| Dream enacting | -0.003 | 0.525 |  | -0.003 | 0.531 |  | -0.001 | 0.844 |
| Sleep is disturbed | 0.001 | 0.780 |  | -0.026 | 0.418 |  | 0.005 | 0.553 |

HC: Healthy Control; NfL: Neurofilament light chain; RBD: Rapid eye movement sleep Behavior Disorder; RBDSQ: RBD Screening Questionnaire

†: adjusted for age, sex and years of education;

| **Table S12. Associations of daytime sleepiness with serum NfL levels in HCs in longitudinal study** | | | | | | | | |
| --- | --- | --- | --- | --- | --- | --- | --- | --- |
| **Sleep characteristics** | **HC** | |  | **Healthy Male** | |  | **Healthy Female** | |
|  | **β** | **P^†^** |  | **β** | **P^†^** |  | **β** | **P^†^** |
| Total score of ESS | -0.001 | 0.172 |  | -0.002 | 0.024 |  | 0.001 | 0.610 |
| Whether had EDS (total score of ESS ≥ 10) | <-0.001 | 0.950 |  | 0.002 | 0.836 |  | -0.011 | 0.401 |
| Possibilities of daytime sleepiness when sitting and reading | -0.002 | 0.421 |  | -0.005 | 0.169 |  | 0.001 | 0.823 |
| Possibilities of daytime sleepiness when watching TV | -0.001 | 0.569 |  | -0.003 | 0.455 |  | 0.001 | 0.732 |
| Possibilities of daytime sleepiness when sitting, inactive in a public place | -0.004 | 0.181 |  | -0.006 | 0.085 |  | -0.001 | 0.815 |
| Possibilities of daytime sleepiness when one as a passenger stays in a car for an hour without a break | -0.002 | 0.498 |  | -0.005 | 0.101 |  | 0.003 | 0.384 |
| Possibilities of daytime sleepiness when lying down to rest in the afternoon | -0.003 | 0.196 |  | -0.006 | 0.015 |  | 0.001 | 0.690 |
| Possibilities of daytime sleepiness when sitting and talking to someone | 0.002 | 0.921 |  | -0.001 | 0.950 |  | - | **-** |
| Possibilities of daytime sleepiness when sitting quietly after lunch | -0.003 | 0.257 |  | -0.005 | 0.155 |  | -0.002 | 0.771 |
| Possibilities of daytime sleepiness in a car, while stopped in traffic | <-0.001 | 0.975 |  | -0.004 | 0.479 |  | 0.022 | 0.228 |

HC: Healthy Control; NfL: Neurofilament light chain; ESS: Epworth Sleepiness Scale; EDS: excessive daytime sleepiness

†: adjusted for age, sex and years of education;

| **Table S13. Associations between change rates of daytime sleepiness and changes of serum NfL in PD individuals** | | | | | | | | |
| --- | --- | --- | --- | --- | --- | --- | --- | --- |
| **Sleep characteristics** | **PD** | |  | **PD Male** | |  | **PD Female** | |
|  | **β** | **P^†^** |  | **β** | **P^†^** |  | **β** | **P^†^** |
| Total score of ESS | 0.001 | 0.267 |  | -0.001 | 0.712 |  | 0.004 | **0.033** |
| Possibilities of daytime sleepiness when sitting and reading | 0.019 | 0.055 |  | 0.012 | 0.361 |  | 0.029 | 0.060 |
| Possibilities of daytime sleepiness when watching TV | -0.002 | 0.856 |  | -0.014 | 0.246 |  | 0.016 | 0.246 |
| Possibilities of daytime sleepiness when sitting inactive in public places | 0.012 | 0.272 |  | -0.001 | 0.949 |  | 0.036 | **0.048** |
| Possibilities of daytime sleepiness when one as a passenger stays in a car for an hour without a break | 0.006 | 0.641 |  | 0.017 | 0.305 |  | -0.013 | 0.540 |
| Possibilities of daytime sleepiness when lying down to rest in the afternoon | -0.001 | 0.884 |  | -0.015 | 0.142 |  | 0.017 | 0.142 |
| Possibilities of daytime sleepiness when sitting and talking to someone | 0.015 | 0.071 |  | 0.008 | 0.459 |  | 0.031 | **0.032** |
| Possibilities of daytime sleepiness when sitting quietly after lunch | 0.001 | 0.943 |  | -0.019 | 0.073 |  | 0.030 | **0.016** |
| Possibilities of daytime sleepiness in a car, while stopped in traffic | 0.012 | 0.165 |  | 0.001 | 0.919 |  | 0.033 | **0.021** |

PD: Parkinson’s disease; NfL: Neurofilament light chain; ESS: Epworth Sleepiness Scale;

†: adjusted for age, sex, years of education and and disease duration; Bold text: significant results

| **Table S14. Associations between change rates of daytime sleepiness and changes of serum NfL in Prodromal PD individuals** | | | | | | | | |
| --- | --- | --- | --- | --- | --- | --- | --- | --- |
| **Sleep characteristics** | **Prodromal PD** | |  | **Prodromal PD Male** | |  | **Prodromal PD Female** | |
|  | **β** | **P^†^** |  | **β** | **P^†^** |  | **β** | **P^†^** |
| Total score of ESS | 0.002 | 0.187 |  | 0.002 | 0.398 |  | 0.001 | 0.605 |
| Possibilities of daytime sleepiness when sitting and reading | -0.022 | **0.071** |  | -0.039 | 0.036 |  | -0.002 | 0.919 |
| Possibilities of daytime sleepiness when watching TV | -0.006 | 0.478 |  | -0.014 | 0.330 |  | -0.007 | 0.503 |
| Possibilities of daytime sleepiness when sitting inactive in public places | 0.008 | 0.565 |  | 0.019 | 0.345 |  | -0.004 | 0.807 |
| Possibilities of daytime sleepiness when one as a passenger stays in a car for an hour without a break | 0.020 | 0.220 |  | 0.021 | 0.412 |  | 0.020 | 0.330 |
| Possibilities of daytime sleepiness when lying down to rest in the afternoon | 0.004 | 0.554 |  | 0.009 | 0.349 |  | -0.004 | 0.653 |
| Possibilities of daytime sleepiness when sitting and talking to someone | 0.021 | **0.018** |  | 0.018 | 0.168 |  | 0.014 | 0.237 |
| Possibilities of daytime sleepiness when sitting quietly after lunch | 0.021 | **0.004** |  | 0.020 | 0.087 |  | 0.016 | 0.078 |
| Possibilities of daytime sleepiness in a car, while stopped in traffic | 0.028 | **0.001** |  | 0.020 | 0.065 |  | 0.039 | **0.024** |

PD: Parkinson’s disease; NfL: Neurofilament light chain; ESS: Epworth Sleepiness Scale;

†: adjusted for age, sex, years of education and diagnoses (RBD, hyposmia and and genetic mutation); Bold text: significant results

| **Table S15. Associations between change rates of daytime sleepiness and changes of serum NfL in HCs** | | | | | | | | |
| --- | --- | --- | --- | --- | --- | --- | --- | --- |
| **Sleep characteristics** | **HC** | |  | **Healthy Male** | |  | **Healthy Female** | |
|  | **β** | **P^†^** |  | **β** | **P^†^** |  | **β** | **P^†^** |
| Total score of ESS | 0.001 | 0.847 |  | <0.001 | 0.996 |  | 0.001 | 0.824 |
| Possibilities of daytime sleepiness when sitting and reading | 0.003 | 0.877 |  | 0.005 | 0.834 |  | -0.012 | 0.754 |
| Possibilities of daytime sleepiness when watching TV | -0.012 | 0.512 |  | -0.003 | 0.891 |  | -0.040 | 0.211 |
| Possibilities of daytime sleepiness when sitting inactive in public places | -0.004 | 0.867 |  | -0.009 | 0.724 |  | -0.005 | 0.921 |
| Possibilities of daytime sleepiness when one as a passenger stays in a car for an hour without a break | 0.023 | 0.370 |  | 0.040 | 0.197 |  | 0.005 | 0.908 |
| Possibilities of daytime sleepiness when lying down to rest in the afternoon | 0.015 | 0.297 |  | 0.018 | 0.349 |  | 0.014 | 0.487 |
| Possibilities of daytime sleepiness when sitting and talking to someone | -0.024 | 0.505 |  | -0.060 | 0.103 |  | 0.193 | 0.066 |
| Possibilities of daytime sleepiness when sitting quietly after lunch | -0.006 | 0.739 |  | -0.018 | 0.356 |  | 0.025 | 0.432 |
| Possibilities of daytime sleepiness in a car, while stopped in traffic | -0.028 | 0.420 |  | -0.052 | 0.169 |  | 0.092 | 0.279 |

HC: Healthy control; NfL: Neurofilament light chain; ESS: Epworth Sleepiness Scale;

†: adjusted for age, sex and years of education;
